# Alginate Sphere-Based Soft Actuators

**DOI:** 10.3390/gels11060432

**Published:** 2025-06-05

**Authors:** Umme Salma Khanam, Hyeon Teak Jeong, Rahim Mutlu, Shazed Aziz

**Affiliations:** 1 School of Life and Medical Sciences, University of Hertfordshire, Hatfield AL10 9AB, UK; khanamummesalma@gmail.com; 2 Division of Energy Engineering, Daejin University, Pocheon 11159, Republic of Korea; jht4321@daejin.ac.kr; 3 Faculty of Science and Engineering, Southern Cross University, Lismore, NSW 2480, Australia; 4 School of Chemical Engineering, The University of Queensland, Brisbane, QLD 4072, Australia

**Keywords:** alginate, hydrogel sphere, smart materials, stimuli-responsive polymers, soft actuators

## Abstract

Alginate hydrogels offer distinct advantages as ionically crosslinked, biocompatible networks that can be shaped into spherical beads with high compositional flexibility. These spherical architectures provide isotropic geometry, modularity and the capacity for encapsulation, making them ideal platforms for scalable, stimuli-responsive actuation. Their ability to respond to thermal, magnetic, electrical, optical and chemical stimuli has enabled applications in targeted delivery, artificial muscles, microrobotics and environmental interfaces. This review examines recent advances in alginate sphere-based actuators, focusing on fabrication methods such as droplet microfluidics, coaxial flow and functional surface patterning, and strategies for introducing multi-stimuli responsiveness using smart polymers, nanoparticles and biologically active components. Actuation behaviours are understood and correlated with physical mechanisms including swelling kinetics, photothermal effects and the field-induced torque, supported by analytical and multiphysics models. Their demonstrated functionalities include shape transformation, locomotion and mechano-optical feedback. The review concludes with an outlook on the existing limitations, such as the material stability, cyclic durability and integration complexity, and proposes future directions toward the development of autonomous, multifunctional soft systems.

## 1. Introduction

Soft actuators derived from hydrogels have emerged as a compelling class of materials that enable adaptive, compliant motion in response to diverse stimuli such as the temperature, pH, light, magnetic fields and electrical signals [1,2,3,4,5]. Their hydrated polymer networks allow them to mimic the mechanical softness of biological tissues while offering responsiveness that is programmable through their chemistry and structure [6,7,8]. This convergence of biological affinity, mechanical compliance and stimuli-coupled deformation has placed hydrogels at the forefront of next-generation applications in soft robotics, biomedical devices and smart environmental systems [9,10,11,12].

Among hydrogel materials, alginate offers unique advantages rooted in its ionic crosslinking mechanism, aqueous processability and compatibility with both biological and synthetic environments [13,14,15,16]. Derived from brown seaweed and rich in carboxylate groups, alginate forms mechanically stable networks via divalent ion coordination, most notably with calcium ions as described by the well-characterised “egg-box” model [17]. This reversible and tuneable gelation process enables the rapid fabrication of soft structures that can swell, contract, degrade or morph in controlled ways. While traditional studies have focused on planar or cylindrical geometries for hydrogel actuators, spherical alginate architectures are now emerging as a distinct and promising subclass. Their isotropic symmetry, ease of fabrication using droplet microfluidics or centrifugal casting and capacity to encapsulate active or living agents open new pathways for decentralised, modular actuation. Moreover, these spheres can be tailored to deform anisotropically when embedded with directional fillers, structured shells or asymmetric stimuli-responsive domains [18,19,20,21].

Recent advances have demonstrated that alginate spheres can be functionalised to exhibit photothermal, magnetic, electroactive and thermoresponsive behaviours, enabling actuation that is wireless, spatially resolved and highly programmable [22,23]. The potential of these microspheres is now being explored for applications including light-controlled microrobots, magnetic cell delivery platforms, electro-responsive artificial muscles and environmentally triggered drug release systems [24,25,26,27]. However, despite this rapid progress, the field lacks a coherent framework that links material design, fabrication techniques, modelling approaches and system-level integration.

This review synthesises the state of the art in alginate sphere-based soft actuators, providing a unified perspective that connects their material foundations with functional applications. We begin by examining fabrication strategies that span from simple dripping to advanced coaxial and layer-by-layer methods, followed by a detailed survey of functionalisation routes that impart external field responsiveness. The role of alginate sphere’s architecture, both intrinsic (e.g., porosity, anisotropy) and extrinsic (e.g., embedding in braids or shells), is evaluated in translating local deformation into a macroscopic mechanical output. To support rational design, we also outline the fundamental physical models governing stimuli–response relationships, including swelling kinetics, thermomechanical coupling, magnetic guidance and light-driven deformation. These models enable the predictive design of actuation profiles and support the inverse engineering of responsive behaviours based on functional requirements.

The significance of this review lies in studying alginate spheres not merely as soft materials but as programmable units of function, each with the potential to sense, actuate and adapt when properly integrated into structured systems. We propose a strategic roadmap, supported by a cross-domain maturity analysis, that outlines the developmental priorities for translating these microscale actuators into intelligent, autonomous platforms. Through a detailed exploration of their material logic, functional responsiveness and architectural integration, this review finds alginate spheres to be a foundational building block for intelligent soft materials with real-world utility.

## 2. Fabrication Strategies for Alginate Spheres

**Figure 1:**
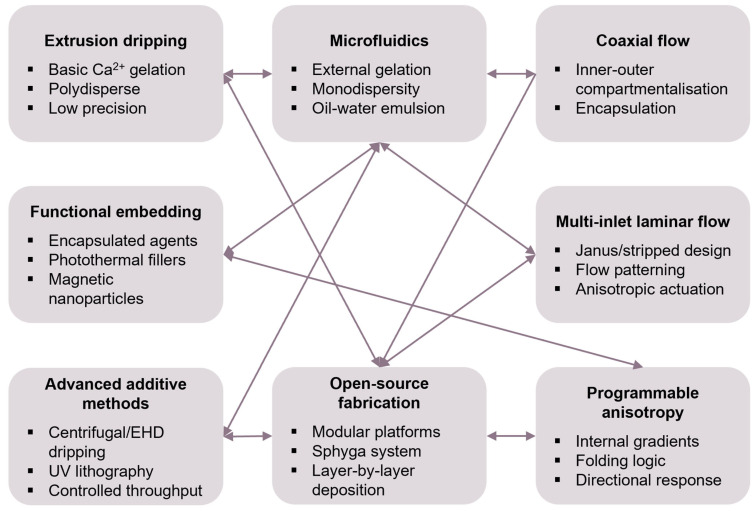
Fabrication strategies and evolution pathways for alginate sphere-based actuators. Arrows indicate logical transitions and/or relationships between methods and their role in realising programmable, multifunctional soft actuators.

The fabrication of alginate spheres as functional building blocks for soft actuators requires the integration of polymer chemistry, microfluidic engineering and stimuli-responsive material science [28]. The ability of alginate to rapidly form hydrogels via ionic crosslinking with divalent cations such as Ca^2+^ enables the production of mechanically stable, highly hydrated networks with adjustable porosity and mechanical stiffness [29]. This gelation, governed by the “egg-box” coordination between calcium ions and guluronic acid residues, provides the structural foundation for embedding actuation capabilities, ranging from thermal expansion to magnetic reconfiguration, within spherical microarchitectures [30]. As illustrated in Figure 1, the fabrication landscape has evolved from static ionotropic methods to modular, flow-programmable platforms capable of engineering anisotropic, multifunctional actuators.

Initial fabrication approaches, such as gravitational dripping or manual extrusion into calcium baths, offered operational simplicity but resulted in broad size distributions and limited control over the internal structure [31]. These limitations restrict their use in applications demanding precise mechanical and functional properties. To address this, microfluidic techniques, particularly droplet-based systems using T-junctions and flow-focusing geometries, have emerged as a breakthrough, enabling the generation of monodisperse alginate emulsions and controlled gelation via interfacial calcium diffusion [32,33,34]. These methods not only enhance the size uniformity but also allow for the encapsulation of functional agents with spatial precision.

Advanced implementations of microfluidics have enabled the fabrication of complex architectures such as core–shell spheres [35,36,37], Janus particles [38,39,40] and compartmentalised beads [19,41,42]. Coaxial flow systems, using nested capillaries, allow for the independent manipulation of the inner and outer phases, supporting multilayered actuation profiles or dual-mode release behaviours [43]. Multi-inlet laminar flow systems further enable directional heterogeneity, with the anisotropic alignment of magnetic particles or stimuli-sensitive domains translating into programmable shape changes [44,45,46]. These structural nuances are critical in realising targeted deformation, directional locomotion and zonal release within soft robotic and biomedical contexts.

Sequential gelation and post-processing steps, such as surface grafting, in situ polymerisation and photopatterning, have expanded the compositional and functional complexity of alginate spheres [47,48,49]. These methods allow for the integration of thermoresponsive (e.g., poly(N-isopropylacrylamide) [50,51], photothermal (e.g., graphene oxide) [52,53] or electroactive (e.g., polycarbazole) [54,55] domains that respond to specific external stimuli. Recent innovations have also highlighted the potential of multi-responsive hydrogels such as alginate-g-P(NIPAm-co-NDEAm), which exhibit synergistic swelling behaviours under thermal, pH and optical stimuli [56]. Other approaches leverage magnetic guidance using Fe_3_O_4_ nanoparticles or aligned nanorods [57], enabling locomotion in constrained environments. These examples demonstrate the functional versatility that emerges from controlling the microscale structure during bead formation. Importantly, the advent of open-source and benchtop platforms like Sphyga has democratised access to programmable bead fabrication, allowing researchers to tune parameters such as the flow rate, nozzle configuration and crosslinking kinetics in real time [58]. These developments are enabling the wider adoption of complex actuator design in non-specialist laboratories.

Despite significant advancements in the fabrication of alginate spheres, scalability remains a central challenge. While techniques such as electrohydrodynamic dripping, centrifugal casting and pressure-assisted extrusion have demonstrated potential for increasing the throughput while preserving control over the bead morphology, several critical issues persist. These include batch-to-batch variability, incomplete crosslinking and the aggregation of functional fillers, all of which undermine the performance reproducibility. Addressing these limitations through systematic investigation is essential, particularly for applications that demand long-term stability and reliability, such as in vivo therapeutic delivery and environmental deployment.

## 3. Functionalisation Approaches

To transform alginate spheres from passive gel matrices into responsive soft actuators, functionalisation is both essential and strategically transformative. This process involves integrating stimuli-responsive components, tailoring the internal polymer network or modifying the surface to introduce actuation triggers such as the temperature, magnetic fields, electric signals, the pH or light [59,60,61,62,63,64]. The intrinsic chemistry of alginate, i.e., a carboxyl-rich backbone capable of ionic crosslinking, offers a versatile scaffold for diverse functionalisation strategies that are chemically stable, biocompatible and modular [65].

**Figure 2:**
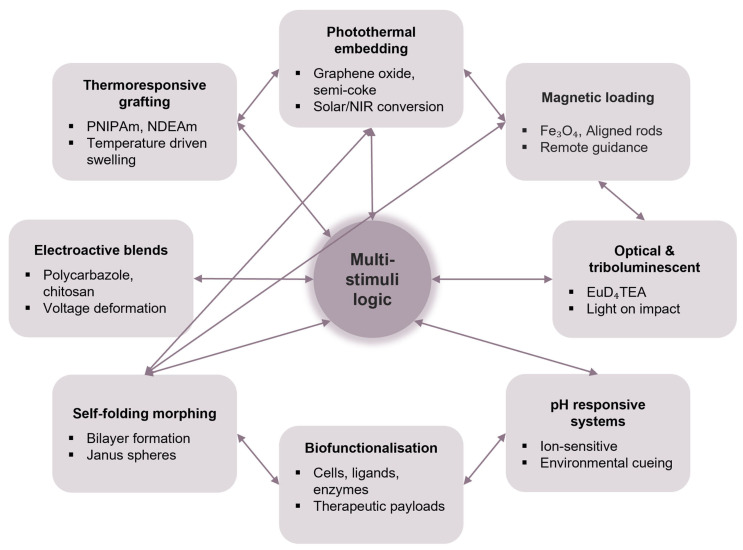
Functionalisation strategies for and integrated capabilities of alginate sphere-based soft actuators.

These functionalisation strategies are critically summarised in Figure 2, which maps out major integration modes, such as thermoresponsive grafting [66,67,68,69], magnetic loading [23,70,71,72], electroactive blending [55,73,74] and biofunctionalisation [75,76], alongside the associated stimuli and resulting actuation behaviours. The diagram also highlights how individual modalities can be synergistically combined into programmable, multi-stimuli-responsive architectures [77]. Rather than being limited to single-mode swelling, alginate spheres have evolved into sophisticated microactuators capable of reversible bending, autonomous locomotion, remote guidance and environmental feedback. The rational incorporation of smart polymers, field-responsive particles and bioactive agents positions these spheres as a modular and scalable platform for advanced soft robotic applications. However, challenges such as their long-term functional stability, the leaching of embedded components and mechanical fatigue under repeated actuation cycles remain critical bottlenecks that demand systematic material- and systems-level optimisation.

One of the most prominent approaches for functionalising alginate spheres involves thermoresponsive modification using poly(N-isopropylacrylamide) (PNIPAm) and its copolymers [78,79,80,81,82,83]. PNIPAm hydrogels exhibit a sharp volume phase transition near physiological temperatures (~32 °C), which allows for reversible swelling–deswelling behaviour [84,85,86]. By grafting PNIPAm or copolymerising it with N,N-diethylacrylamide (NDEAm), researchers have developed alginate-based beads capable of finely tuned volume changes in response to the temperature and light [78,79]. The inclusion of photothermal agents such as semi-coke particles further extends their responsiveness to solar or NIR light, enabling the use of environmental triggers to drive release or deformation [87,88,89]. These systems have found promising applications in precision agriculture, where the on-demand delivery of agrochemicals is critical.

Magnetic actuation presents another powerful mode of functionalisation. By incorporating iron oxide nanoparticles or magnetically aligned nanorods into alginate beads, the resultant microspheres gain remote manipulability via external magnetic fields [23,90,91,92,93]. This capability enables complex robotic behaviours such as rolling, tumbling, orientation control and cargo transport at small scales. Notably, anisotropic loading or Janus structuring enhances the torque and directional control, allowing the beads to function as mobile microrobots in both fluid and surface environments. Self-folding bilayers composed of magnetic alginate and photothermal hydrogels have further demonstrated programmable shape morphing, enabling bead-based devices to open, close or reconfigure themselves on command [94].

Electrically responsive hydrogels have also emerged by blending alginate with electroactive polymers such as polycarbazole [54]. These blends exhibit reversible bending and deformation under an applied voltage, suitable for low-voltage actuation in aqueous environments. Similarly, chitosan–alginate systems fabricated via electrodeposition offer pH-sensitive gelation and electro-responsive swelling, enabling spatially controlled drug release and shape transformation. The electrodeposition process also permits the conformal coating of complex microscale geometries, expanding the design space for embedded hydrogel devices.

Light-emitting or optically responsive beads represent a unique class of functionalised alginate systems [95]. Composite microspheres embedded with triboluminescent EuD_4_TEA crystals generate light upon a mechanical impact, offering mechano-optical feedback suitable for damage sensing or interactive microrobotics [96]. The swelling of the alginate matrix also modulates the luminescent signal, coupling an optical response with structural hydration. Such dual-mode actuation and sensing may offer new paradigms in interactive soft systems [97]. Environmental responsiveness, particularly pH sensitivity, is another widely leveraged property. The intrinsic ionisable groups of alginate make it susceptible to environmental pH changes, which alter the crosslinking density and network swelling. This property has been further enhanced in multi-responsive systems, where pH sensitivity is coupled with thermal or photothermal cues to enable programmable substance release [56]. In environmental applications, this principle has enabled the design of pollutant-sensing or controlled-release devices, particularly for use in soil, water purification or agricultural delivery systems. Finally, biofunctionalisation with biological ligands, enzymes or cells is gaining interest for therapeutic and biomedical applications [65,98,99,100,101]. Functional beads containing stem cells or loaded with therapeutic payloads have been designed for targeted delivery, minimally invasive surgery and use as tissue engineering scaffolds [94,102]. The gentle gelation conditions of alginate and its biocompatibility make it highly suitable for integration with sensitive biological elements without compromising their viability or activity.

## 4. Actuation Mechanisms and Performance

The functional diversity of alginate sphere-based actuators arises from the interplay of hydrogel chemistry, stimuli–response coupling and embedded material engineering. These actuators leverage fundamental physical mechanisms, such as osmosis, thermal expansion, magnetic torque, electrochemical migration and photothermal conversion, to produce mechanical deformation. The underlying alginate matrix, composed of guluronic and mannuronic acid residues, forms ionically crosslinked “egg-box” domains with divalent cations like Ca^2+^. This physically crosslinked network supports a high water uptake, reversible swelling and mechanical compliance. However, the performance envelope of these systems depends on the integration of additional responsive chemistries, mechanical structuring and functional fillers, as highlighted in Table 1.

Thermoresponsive systems represent one of the most extensively studied classes of alginate actuators. Here, PNIPAm or its copolymers undergo coil-to-globule transitions at 32 °C, collapsing the polymer network and expelling water. When grafted onto the alginate backbone or dispersed within the gel, this behaviour enables a substantial volume change. Notably, copolymerisation with NDEAm allows for the fine-tuning of the lower critical solution temperature (LCST), while blending with photothermal fillers such as semi-coke imparts solar or NIR responsiveness [78,79]. From a physical chemistry perspective, the actuation is driven by the entropic collapse of hydrated polymer chains, with the transition kinetics governed by the heat transfer and water mobility. Actuation strains of up to 50% have been reported [56], though the response time remains in the 1–3 min range due to slow thermal diffusion through hydrated matrices. These systems hold promise for use in precision agriculture and soft dispensing applications, where the need for a high strain magnitude outweighs the need for rapid responsiveness.

In contrast, magnetic actuation exploits the torque and translational forces applied to embedded Fe_3_O_4_ or aligned magnetic nanostructures. These particles introduce magnetic anisotropy and rotational dynamics under an applied field. The advantage here lies in fast, wireless control over the bead orientation and motion, with sub-second response times reported [57,103]. The physical basis lies in the torque generated by aligning dipole moments with the external magnetic vector. Unlike thermoresponsive systems, magnetic actuation does not rely on solvent diffusion and is unaffected by environmental hydration. These systems are particularly effective in biomedical microrobotics and fluidic navigation, though their volumetric strain is modest (~10–12%). A limitation, however, is the non-deformational nature of the response, which enables locomotion but not substantial shape changes.

Swelling-based actuation, the mechanism most chemically intrinsic to alginate, is driven by osmotic gradients across the hydrogel network [104,105]. A water influx increases the network entropy, causing polymer chains to extend. When deployed in structured forms, such as braided sleeves, this radial expansion can be converted into axial contraction, resembling the behaviour of McKibben actuators [50,106]. In our view, this architecture best exemplifies the coupling between soft material chemistry and macroscopic mechanics. Forces of up to 5–6 N and displacements of 7–8% have been observed. However, the high water content and large diffusion lengths involved result in long actuation time scales. For real-time robotics, such sluggishness is a bottleneck unless mitigated through miniaturisation or by the structural porosity.

**Table 1:** Actuation mechanism and comparative performance of alginate sphere-based soft actuators.

System/Material	Stimulus	Max Actuation Strain (%)	Blocking Force (N)	Response Time	Actuation Mode	Application Domain	Reversibility/Cyclic Use	Reference
Alginate-g-P(NIPAm-co-NDEAm)/SC	Temperature, pH, light	~50%	Not reported	1–3 min	Volumetric swelling/deswelling	Controlled agrochemical release	High, multi-cycle-tested	[56]
Magnetic alginate beads	Magnetic field	~10%	Not reported	Instantaneous	Magnetic rotation/translation	Targeted drug delivery	High, magnetic field-controlled	[57,107,108]
Braided hydrogel muscle	Temperature (cooling from 60 °C)	7–8%	5–6 N	Slow (minutes)	Contraction due to swelling	Artificial muscles/soft robotics	Moderate (fatigue observed)	[50]
Electroactive alginate–polycarbazole	Electric field (low voltage)	1–2%	Low (µN-mN)	Seconds	Voltage-induced deformation	Electroactive sensing or actuation	Limited (electrochemical fatigue)	[55]
Photothermal GO–alginate bilayers	NIR light	15–20%	Not reported	Fast (~seconds)	Bending/folding due to heating	Microrobotics, biomedical folding	Good, NIR-cycled	[94]
Magnetic alginate micromotors	Magnetic field	~12%	Not reported	Sub-second rotation	Magnetic propulsion	Remote-controlled microswimmers	Yes, in fluid environment	[103,109]
pH-responsive Ca–alginate beads	pH variation (acidic)	5–10%	Not applicable	2–5 min	Swelling and gel softening	Environmental remediation	Yes, but pH-limited	[110,111]
Triboluminescent EuD4TEA–alginate beads	Mechanical impact	Swelling-dependent	Not applicable	Instantaneous flash	Optical emission due to deformation	Mechanical sensing and diagnostics	No, single flash	[96]
Piezoelectric alginate microspheres	Electric field (piezoelectric)	0.5–1%	Very low	Milliseconds	Electric field-induced deformation	Biosignal-responsive systems	Yes, piezoelectric loop	[112]

Electro-responsive actuation introduces electronic control through the blending of alginate with conductive polymers like polycarbazole. When a voltage is applied, ion migration and localised redox reactions alter the swelling behaviour of the polymer network. These systems operate on principles analogous to those of ionic electroactive polymers (EAPs) and enable bidirectional bending or strain upon low-voltage activation. However, electrochemical side reactions, ion depletion and fatigue limit their long-term use. With strains in the 1–2% range and forces in the μN–mN domain, they are better suited for microscale actuation and sensing. From a materials perspective, the challenge lies in stabilising the redox-active domains within an ionically crosslinked network, which itself is sensitive to the charge density and electrostatic breakdown. Photothermal actuation uses embedded light-absorbing fillers, such as graphene oxide, carbon black or semi-coke, to convert light into heat, initiating thermally induced deformation [55,113,114]. This approach is notable for offering wireless, spatially targeted control with a fast response. The local photothermal effect follows classical Fourier heat diffusion, while the resultant actuation relies on the presence of embedded thermoresponsive polymers. This mechanism is particularly effective in soft microrobots that require untethered control in aqueous environments [94]. However, repeated cycling can induce localised heating stress and affect the stability of the hydrogel network.

Table 1 also highlights additional mechanisms such as pH-responsive actuation for environmental remediation, triboluminescence for mechanical sensing and piezoelectric responses for bioelectric interfaces. These niche systems extend the scope of application but currently lack optimisation in terms of their performance metrics. For example, pH-sensitive beads can offer 5–10% swelling and softening, useful for adsorption or use in smart filtration systems [110,111]. Triboluminescent beads emit light upon an impact due to EuD4TEA crystal fractures but are not suitable for repeatable mechanical work [96]. From a performance outlook, each actuation mechanism involves trade-offs among the strain, force, response time and operational stability. Thermal systems maximise the volumetric strain but respond slowly. Magnetic systems offer fast actuation and mobility but with limited deformation. Electroactive gels provide programmability but suffer from a low mechanical output. Swelling-based actuators generate useful forces but lack speed. Photothermal systems sit at a functional crossroad, balancing remote control, the strain and speed, making them particularly versatile.

However, the current literature lacks standardisation in its reporting methods, making cross-comparisons difficult. Metrics such as the blocking force, cycle life, energy density and fatigue resistance have been inconsistently reported. We believe the field would benefit from benchmarking protocols analogous to those used in dielectric elastomers or shape memory alloys. Moreover, the long-term behaviour under cyclic conditions, osmotic fatigue and environmental variability (e.g., ionic strength, pH drift) remain underexplored. Looking forward, there is strong potential in hybrid systems that couple multiple actuation modalities, such as photothermal–magnetic or pH–electro-responsive constructs. Integrating sensing capabilities (strain, pressure, chemical) with actuation in a single bead could realise the creation of autonomous, decentralised robotic units. The computational modelling of stimuli–response coupling, especially using finite element simulations or multiphysics models, will be critical for the design of next-generation systems.

## 5. Design Logic and Actuation Modelling

### 5.1. Integration into Functional Architectures

While the intrinsic actuation capabilities of alginate-based spheres are diverse and promising, their effective deployment in soft robotic systems requires their thoughtful integration into larger functional architectures. The translation of their material-scale responsiveness into meaningful robotic motion or adaptive mechanical work depends on how these hydrogel spheres are structured, constrained and interfaced with the surrounding components. This integration bridges the gap between material science and device engineering and ultimately determines the system-level performance, reliability and utility.

One of the most conceptually mature and mechanically effective designs is braided artificial muscle, wherein alginate spheres are embedded within a mesh sleeve or tubular braid. This configuration operates on the McKibben principle: when the hydrogel spheres swell (e.g., due to temperature shifts and water absorption), they generate radial expansion, which, due to the geometric constraints of the braid, is converted into axial contraction. This ingenious structural transformation amplifies the modest swelling strain of individual beads into collective linear motion [50]. Modular sphere arrays offer a strategy for scalable and reconfigurable actuation. Distributed across a soft matrix, spheres can be selectively activated by light, the temperature or pH gradients. Achieving precise control in these systems depends not only on the material design but also on the use of predictive models that relate the stimulus input to the deformation output.

### 5.2. Fundamental Actuation Models

At the core of modelling hydrophilic polymer systems is the Flory–Rehner theory, which describes the equilibrium swelling pressure Π inside a hydrogel [115]:

(1) $$\Pi =-RT\;\left(\phi /\nu \right)[\ln\left(1-\phi \right)+\phi +\chi \phi^{2}]$$

This equation captures the entropic and enthalpic contributions to mixing between the polymer and solvent. Here, ϕ is the polymer volume fraction, ν denotes the molar volume of the solvent, χ defines the polymer–solvent interaction parameter, R is the universal gas constant and T denotes the temperature. Typically, the swelling pressure drives the expansion of alginate spheres in aqueous environments, forming the mechanical basis for actuation.

Swelling does not occur indefinitely; it is resisted by the elastic retraction of the polymer network. For hydrogels such as alginate crosslinked with calcium ions, the elastic stress can be modelled using the following neo-Hookean approximation [116,117]:

(2) $$\sigma_{elastic}=G\left(\lambda^{2}-\lambda^{-1}\right)$$

Here, G is the shear modulus of the network and λ is the stretch ratio. At equilibrium, the swelling pressure balances the elastic stress (Π = $\sigma_{elastic}$), defining the final bead size and available actuation strain. This coupling of osmotic and elastic terms is fundamental to all hydrogel-based actuators. However, swelling is not instantaneous. Its time dependence is governed by Fickian diffusion. For a spherical bead, the radial growth can be approximated as [118,119]

(3) $$R\left(T\right)/R_{0}\approx 1+\left(6/\pi^{2}\right)\exp\left(-D{.\pi }^{2}.t/{R_{0}}^{2}\right)$$

where D is the diffusion coefficient and $R_{0}$ the initial radius. This expression shows that the swelling time scales with the square of the bead size, explaining the sluggish response observed in large actuators. This also justifies efforts to miniaturise spheres or incorporate porosity for a fast response. In structured systems like braided actuators, the swelling pressure generates axial contraction due to geometric constraints. The force output of such a muscle can be approximated by [120]

(4) $$F=PA\left(1-\cos^{2}\theta \right)$$

where P is the internal pressure (estimated from Equation (1)), A the internal cross-sectional area of the braid and θ the braid fibre angle. This equation explains how radial swelling becomes an axial force and enables linear contraction. It is directly applicable to McKibben-type hydrogel actuators and aids in predicting the stroke length and blocking force.

### 5.3. Stimuli-Induced Actuation Models

Beyond general swelling-driven designs, alginate-based actuators can be strategically tuned to respond to external stimuli including light, magnetic fields and temperature shifts, enabling a range of controlled, directional and multifunctional behaviours across diverse environments. For instance, photothermal actuation leverages embedded nanomaterials to convert light into heat, triggering temperature-dependent swelling changes [121]. The temperature rise under light exposure is modelled as [122,123]

(5) $$∆T=\left(I.\alpha .t\right)/\left(\rho .C_{p}.d\right)$$

where I is the light intensity, α defines the absorption coefficient of the photothermal filler, ρ is the density, $C_{p}$ denotes the heat capacity and d is the thickness of the material. This informs how fast and how much the material will heat under illumination, which is crucial for systems using PNIPAm-based copolymers or shape-morphing bilayers [94]. For rolling or tumbling microrobots, the magnetic torque that drives movement is expressed by [124,125,126]

(6) τ=m.B.sin⁡θ

where m is the magnetic moment of the sphere and B is the applied field. The response speed is governed by the balance of this torque and the viscous drag in low-Reynolds-number conditions [127,128]:

(7) $$\omega =\left(m.B.\sin\theta \right)/\left(8.\pi .\mu .R^{3}\right)$$

where ω is the angular velocity and μ the fluid viscosity. These relations are critical for controlling the speed and stability of microrobot navigation using magnetic alginate spheres [57]. Bilayer systems, in which differential swelling causes bending, are often modelled using Timoshenko’s theory [129,130]:

(8) $$\kappa =\left[6\left(\varepsilon_{1}-\varepsilon_{2}\right)\right]/\left[h\left(1+E_{1}/E_{2}\right)\right]$$

Here, κ is the curvature, $\varepsilon_{1}$ and $\varepsilon_{2}$ are the strains in each layer, $E_{1}$ and $E_{2}$ define their moduli and h is the total thickness. This model has been useful for designing folding, gripping or wrapping actuators with tuneable shape responses. Programmable systems may also rely on material-encoded logic, where functional responses are embedded directly within the material structure, such that beads are triggered only when a stimulus exceeds a certain threshold [131]. This approach eliminates the need for external computation by using intrinsic material properties (e.g., the crosslinking density, filler concentration or swelling thresholds) to determine when and how actuation occurs, enabling decentralised, stimulus-responsive behaviour, and can be represented as

(9) $$s_{i}=1\;if\;S_{i}>S_{thresh,i},\;else\;0$$

where $S_{i}$ is the binary state of bead i and $S_{thresh,i}$ its embedded threshold value. This expression supports decentralised control and selective actuation within arrays, enabling logic-like responses, embodied intelligence and spatiotemporal patterning.

**Figure 3:**
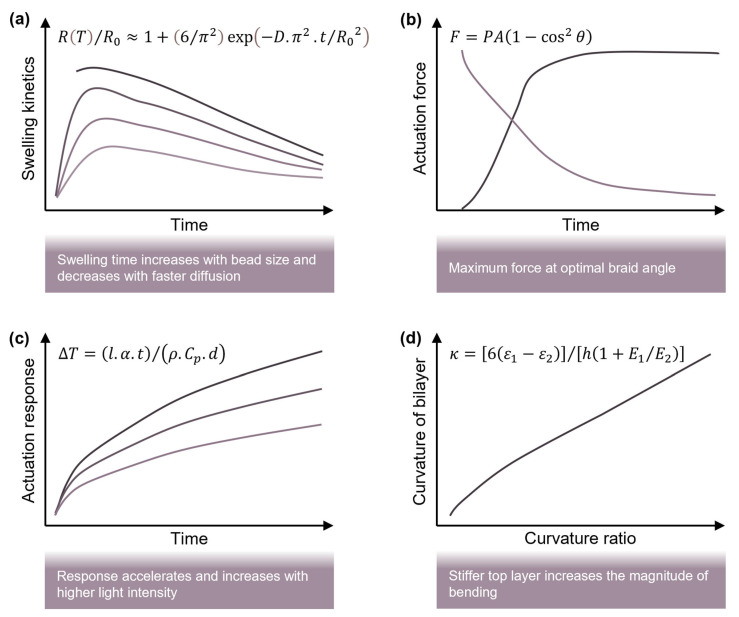
Modelling relationships and design strategies for alginate sphere-based actuators. (**a**) Swelling kinetics model: sphere radius increases over time based on diffusion coefficient D and initial size $R_{0}$, informing design of systems with fast responses. (**b**) Axial force generation in braided architectures: force output varies with internal swelling pressure, braid angle θ and cross-sectional area A, supporting mechanical optimisation of McKibben-type actuators. (**c**) Photothermal actuation: temperature rise in response to increased light intensity (I) and filler absorption coefficient (α) enables programmable, wireless activation in optically triggered systems. (**d**) Bilayer curvature: mismatch in strain and modulus across layers governs bending deformation, useful for shape-morphing and folding actuators.

To consolidate the core modelling relationships that underpin alginate sphere actuation, we schematically map the key physical models (Equations (3)–(5) and (8)) to their controlling parameters, actuation responses and associated design strategies (Figure 3). Figure 3a illustrates the time-dependent swelling behaviour of alginate beads governed by Fickian diffusion (Equation (3)). As the diffusion coefficient (D) increases or the initial bead radius ($R_{0}$) decreases, swelling occurs more rapidly, providing a fast actuation response. This model informs sphere sizing, porosity tuning and network design for a minimal actuation lag. Figure 3b focuses on the mechanical output in braided muscle architectures. Here, the internal swelling pressure is translated into axial contraction through the McKibben principle. Equation (4) relates the axial force to the pressure (P), braid fibre angle (θ) and cross-sectional area (A). The model predicts that moderate angles (~45°) offer the best balance between the stroke length and force output, guiding the design of effective cylindrical or sleeve-type actuators.

Figure 3c captures photothermal actuation dynamics, where localised heating due to absorbed light leads to swelling transitions. The temperature rise (∆T) is described by Equation (5), dependent on the light intensity (I), photothermal efficiency (α), material density (ρ) and thermal diffusivity. This model is critical for wireless activation in soft systems using graphene oxide, semi-coke or metal nanoparticles, where the heat is modulated for both responsiveness and safety. Figure 3d presents the Timoshenko bilayer model (Equation (8)), which governs the curvature (κ) in layered hydrogels exhibiting a mismatched strain and stiffness. The strain mismatch ($\varepsilon_{1}-\varepsilon_{2}$) and modulus ratio ($E_{1}/E_{2}$) determine how sharply and in what direction the system folds or curls. This behaviour underpins programmable geometry in reconfigurable robots, untethered grippers and folding drug carriers.

## 6. Application Demonstrations and Potential

### 6.1. Microrobotic Locomotion and Artificial Muscles

The advancement of alginate sphere-based actuators has enabled their deployment across a wide spectrum of application domains, ranging from microrobotics and soft artificial muscles to therapeutic delivery and bioinspired adaptive materials. Their chemical tunability, hydrogel mechanics and compatibility with stimuli-responsive fillers allow them to undergo programmable deformation, controlled release and environmental adaptation.

In the domain of mobile microrobotics, magnetically aligned nanorods in alginate capsules (MANiACs) have been shown to be capable of rolling and tumbling across soft and planar surfaces under the influence of rotating magnetic fields [57,132]. These microspheres feature internal anisotropy derived from aligned Fe_3_O_4_ nanorods, which allows for precise torque transfer and directional control. Their ability to conform to irregular biological surfaces makes them suitable for surface navigation in diagnostic systems or therapeutic delivery scenarios, where untethered locomotion is crucial. Similarly, thermally driven contraction in McKibben-style artificial muscles can be achieved by embedding PNIPAm–alginate hydrogel spheres within a braided mesh [50]. Upon heating, the embedded beads swell radially, generating internal pressure that translates into axial contraction through the sleeve geometry, validating their integration into soft robotic systems for adaptable gripping or compliant motion. The use of magnetic alginate spheres has been further extended by designing microrobots that combine locomotion and field-triggered cargo delivery [133]. These Janus-structured beads, responsive to oscillating magnetic fields, can be directed through fluidic environments to release encapsulated payloads at precise locations. Their functional asymmetry enhances the torque efficiency and trajectory control, supporting applications in targeted therapy or localised sensing within branching microvascular networks.

### 6.2. Shape Morphing and Biomimetic Resilience

**Figure 4:**
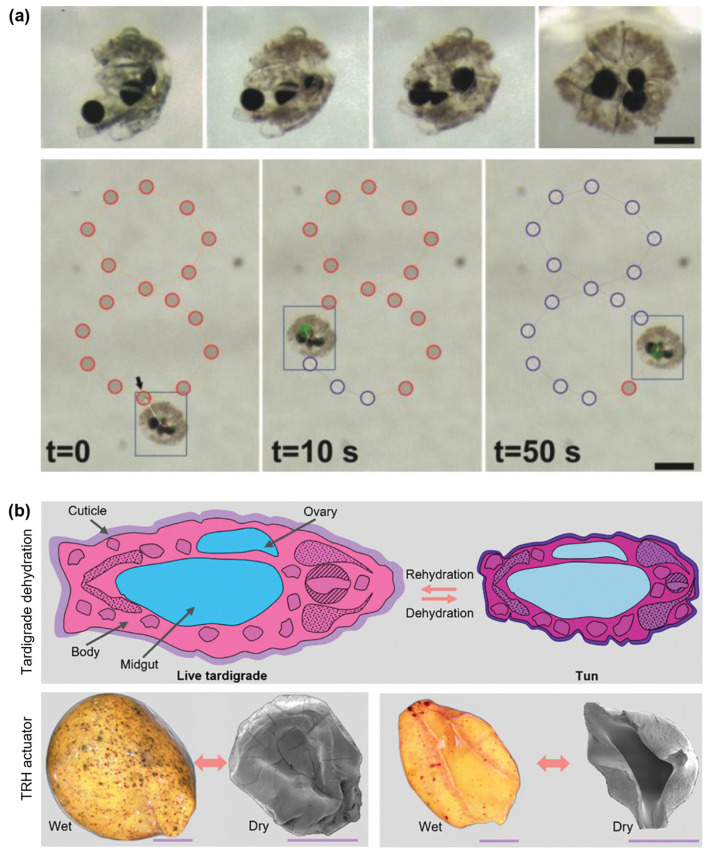
Representative application demonstrations of alginate sphere-based actuators. (**a**) NIR-triggered self-folding Janus microrobots for encapsulation and delivery (scale bar: 200 µm) and microrobot manipulation along a selected route (scale bar: 50 µm). Robot follows preplanned trajectory. Red circles denote target destinations and circles turn blue as robot passes over them [94]. (**b**) Biomorph soft actuators mimicking tardigrade tun formation with microwave-driven contraction (scale bar: 50 µm) [134].

The foundational demonstrations of shape-morphable alginate spheres have paved the way for the development of more complex and multifunctional systems. As shown in Figure 4, several pioneering demonstrations have exemplified this versatility across functionally and biologically relevant contexts. For example, the fabrication of NIR-responsive self-folding microrobots has been demonstrated by engineering Janus alginate structures with photothermal fillers [94]. Upon NIR exposure, differential heating across the bead causes anisotropic swelling and mechanical bending, enabling encapsulation, gripping or shape reconfiguration. These microrobots autonomously change their configuration, enabling light-programmed interactions and the manipulation of soft micro-objects in confined environments (Figure 4a).

Expanding on this concept of biomimetic responsiveness, a novel class of biomorph soft actuators (BSAs) have been developed that emulate tardigrade-like resilience [134]. These hollow alginate spheres, composed of a thermoresponsive PNIPAm hydrogel (TRH) inner core encased within a porous alginate shell, contract under dehydration or microwave heating and re-expand upon rehydration (Figure 4b). Their reversible tun formation enables resilient actuation cycles and survival under extreme chemical, thermal and compressive conditions. Embedded into McKibben-style sleeves, BSAs produced over 9% contraction strain with an accelerated response speed under microwave stimulation, demonstrating their potential for use in high-speed, rugged soft robotic systems or environmental actuators with applications in the removal of heavy metals from industrial effluents.

### 6.3. Biomedical Delivery and Regenerative Applications

**Figure 5:**
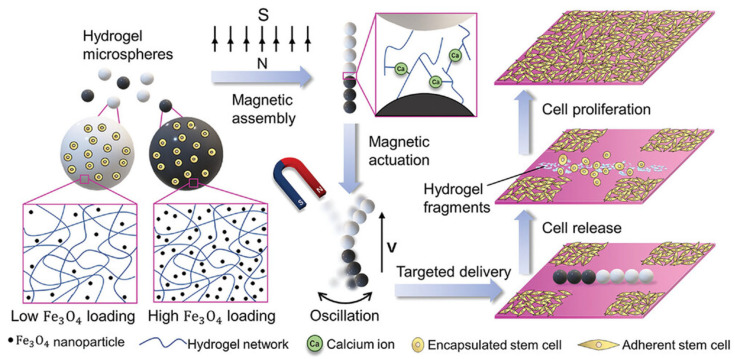
Schematic illustration of a magnetic microsphere scaffold (MMS)-based microrobot designed for targeted stem cell delivery [135].

From a biomedical perspective, the fabrication of a sophisticated microscale delivery platform has been demonstrated based on magnetically steerable alginate microspheres which can transport mesenchymal stem cells (MSCs) to target tissue sites [135]. These microspheres, termed magnetic microsphere scaffolds (MMSs), integrate magnetic nanoparticles within an alginate hydrogel matrix and are loaded with viable MSCs. Under an external oscillating magnetic field, the MMSs self-assemble into a chain-like microrobot structure and exhibit undulatory locomotion reminiscent of flagellar motion (Figure 5). This dynamic actuation mechanism allows the construct to navigate complex biological geometries, such as vascular-like microchannels or tissue gaps, and reach specific sites with spatial precision. Upon arrival at their destination, the microspheres respond to physiological ionic triggers—notably extracellular sodium or phosphate-buffered saline (PBS)—which initiate the partial dissolution of the alginate matrix, thereby releasing the encapsulated stem cells. This dual functionality, i.e., remote guidance and stimuli-responsive cell release, provides a highly controlled delivery system with minimal invasiveness. Its regenerative potential was demonstrated in vitro through scratch wound assays, showing accelerated closure when MMS-delivered MSCs were released at the wound site.

This approach exemplifies how alginate hydrogels can serve as both a scaffold and actuator, leveraging their biocompatibility, ionic responsiveness and capacity for encapsulation. Compared to traditional stem cell injection, which often suffers from low cell retention and non-specific deposition, magnetically actuated hydrogel systems improve the localisation accuracy and mechanical protection during transit. Similar concepts have been reported using magnetically responsive microrobots composed of chitosan or poly(lactic-co-glycolic acid) (PLGA) carriers, but alginate offers distinct advantages in terms of its tuneable porosity, gentle gelation conditions and in vivo biodegradability without harmful degradation by-products [133,136].

### 6.4. Sustainable Actuator Solutions

Alginate is a naturally occurring anionic polysaccharide derived from brown seaweed, offering a compelling combination of sustainability, biocompatibility and environmental degradability. Its gelation in the presence of divalent cations (e.g., Ca^2+^) enables solvent-free, low-energy fabrication under physiologically compatible conditions, making it particularly attractive for use in biomedical and wearable technologies. Importantly, alginate is free from the petrochemical residues and toxic by-products commonly associated with synthetic polymers and can be sourced renewably without competing with food chains or requiring intensive purification steps.

In the context of bioelectronics and soft actuator platforms, spherical alginate-based constructs offer unique functional and environmental advantages over their synthetic counterparts, such as polydimethylsiloxane [137,138], polyacrylamide [139,140] and polyurethane [141,142]. While these synthetic materials often provide superior mechanical durability and thermal resilience, they are typically petroleum-derived, reliant on energy-intensive or solvent-based fabrication processes and non-biodegradable. In contrast, alginate spheres are formed through gentle ionic crosslinking in aqueous media, enabling scalable, solvent-free and low-temperature processing under ambient conditions. This makes them exceptionally well-suited for applications requiring transient operation, disposability or biocompatibility, such as point-of-care diagnostics, implantable delivery platforms and environmentally degradable microrobots. Their geometry further supports modular integration, a high surface area-to-volume ratio for responsive behaviour and compatibility with scalable fabrication methods like droplet microfluidics and centrifugal templating. With their isotropic shape, alginate spheres also offer uniform end-of-life degradation without any toxic residues.

Aligning with circular economy principles, alginate gels are inherently biodegradable and can be engineered for compositional upcycling without compromising the material safety. However, challenges such as variability in natural feedstocks, limited mechanical durability and sensitivity to ionic exchange in physiological environments remain barriers to their widespread application. Current methods, such as blending them with nanocellulose, designing interpenetrating networks or incorporating self-healing moieties, are advancing the utility of alginate while preserving its environmental credentials. As such, future developments should centre on material standardisation, closed-loop biodegradation and integration with sustainable fabrication pipelines to fully realise the potential of alginate as a next-generation platform for intelligent, eco-conscious devices.

## 7. Outlook

Alginate sphere-based actuators are emerging as a versatile and programmable class of soft-matter systems capable of performing complex mechanical and biological tasks. Their adaptability across stimuli, ranging from thermal and magnetic to chemical and electrical, positions them at the frontier of intelligent soft robotics, biomedical engineering and responsive environmental interfaces. These systems integrate chemical responsiveness, mechanical compliance and architectural modularity, allowing for distributed, scalable actuation and sensing. However, their real-world deployment beyond controlled laboratory conditions necessitates the deeper integration of material performance research, systems engineering and computational logic.

Future directions for alginate-based actuators must prioritise the co-design of the material composition, stimuli responsiveness and structural function. The development of multifunctional hydrogels with embedded actuation and sensing capabilities and logic will be a critical step. Achieving this will require innovations in dynamic crosslinking networks, copolymer architectures and anisotropic fillers that enable programmable swelling and directional deformation. Emerging fabrication methods such as coaxial and multiphase microfluidics, digital light processing (DLP) and centrifugal templating are expected to unlock higher-order structural complexity at the microscale and mesoscale levels. Likewise, voxel-wise control over the material placement and functional domains could significantly advance the programmability of deformation patterns and actuation response. From a device perspective, the integration of functionalised alginate spheres into architected structures, such as braided artificial muscles, modular sleeves or microrobotics locomotion systems, will facilitate translation from single-particle actuation to collective system-level behaviour. Incorporating feedback loops, autonomous response logic and embedded environmental coupling will be essential for deploying these systems in dynamic or unstructured environments. Further, convergence with biocompatible electronics and adaptive biointerfaces may open new frontiers in implantable therapeutics and smart diagnostics.

**Figure 6:**
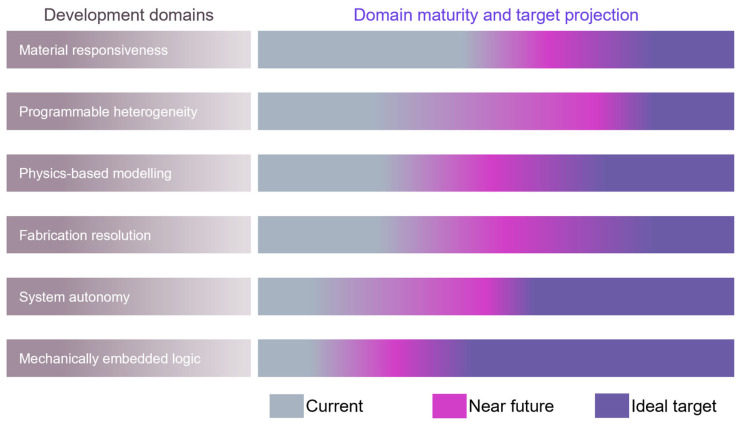
Strategic landscape of alginate actuator development.

Despite the rapid progress in material formulation and actuation demonstrations, the field faces several multi-dimensional challenges that limit alginate spheres’ widespread adoption. As illustrated in Figure 6, six core domains govern the maturity and integration of alginate-based actuator technologies: material responsiveness, programmable heterogeneity, multiphysics modelling, fabrication resolution, system integration autonomy and mechanically embedded logic. These domains are deeply interdependent; for instance, improving the modelling fidelity necessitates the development of well-characterised programmable materials with consistent actuation profiles. System integration autonomy defines the capacity of the actuators to operate independently within larger robotic or biomedical platforms, incorporating elements such as their energy supply, stimulus processing and feedback control without relying on continuous external commands. In parallel, mechanically embedded logic denotes the encoding of functional behaviours, such as directional responses, threshold activation or sequence timing, directly into the material’s structure or composition. This is achieved through strategies like spatial patterning, gradient crosslinking or anisotropic swelling, allowing actuators to execute complex tasks without the need for electronic computation.

Figure 6 also shows the current maturity, near-term developmental outlook and ideal performance targets across these six domains. The material responsiveness currently exhibits moderate maturity, with well-established chemistries for thermal and magnetic actuation but limited robustness under cyclic fatigue and chemical degradation. Programmable heterogeneity and the fabrication resolution score similarly due to the associated challenges in integrating spatially varying material properties at scale. Multiphysics modelling remains limited by abstraction gaps; for example, existing models such as the Flory–Rehner swelling theory and classical elasticity fail to capture the dynamic coupling between chemical kinetics, deformation and environmental stimuli. System-level integration, including embedded sensing capabilities and logic-driven actuation, remains nascent, with most current systems still reliant on external control or sequential activation. The most underdeveloped domain remains mechanically embedded logic, which refers to the encoding of decision-making behaviours into the physical architecture of the actuator itself—this area holds transformative potential but is largely conceptual at present.

To accelerate the advancement of spherical alginate actuators, the following strategic directions are recommended:

▪ Integrate actuation, sensing and mechanical logic into unified composite hydrogels with reconfigurable and adaptive functionality.
▪ Develop real-time, multiphysics modelling platforms that incorporate environmental coupling and deformation-based feedback mechanisms.
▪ Scale high-resolution fabrication strategies capable of encoding spatial heterogeneity and internal actuation logic across large bead or scaffold arrays.
▪ Incorporate adaptive features such as mechanical memory, self-healing or chemo-mechanical conditioning to improve devices’ resilience and operational lifespan.
▪ Transition to using recyclable, biodegradable and bio-derived alginate composites to align with sustainability and circular economy frameworks.

The future of alginate actuator systems will be shaped by their ability to shift from passive soft matter to embodied intelligent systems, where their function, computation and adaptation are inseparable from their material structure. In wearable and soft electronics, spherical alginate-based actuators present a sustainable alternative to synthetic polymers like polyacrylamide and polyurethane, which rely on petrochemical sources and solvent-intensive processing. Formed via aqueous ionic crosslinking under ambient conditions, alginate spheres enable low-energy fabrication with biocompatibility and biodegradability functions. Their isotropic shape supports uniform actuation and rapid, residue-free degradation, which is ideal for transient devices such as diagnostic patches and disposable sensors. While synthetic hydrogels offer greater durability, advances in nanocomposite reinforcement and structural design are closing this gap, making alginate spheres increasingly viable for scalable, eco-friendly applications. Inspired by progress in nanomaterial soft actuators and informed by advances in machine learning-guided material design, we envision a new generation of alginate-based actuators that act, respond and evolve with their environments. In such systems, logic is not embedded in circuits, but in molecular gradients, mechanical thresholds and distributed responsiveness. Realising this vision will require deep interdisciplinarity that will bridge polymer chemistry, data-driven modelling, high-resolution fabrication and system-level design.

## Data Availability

No new data were created or analysed in this study.

## References

[1] Ding M., Jing L., Yang H., Machnicki C.E., Fu X., Li K., Wong I.Y., Chen P.Y. (2020). Multifunctional soft machines based on stimuli-responsive hydrogels: From freestanding hydrogels to smart integrated systems. Mater. Today Adv..

[2] Jiao D.J., Zhu Q.L., Li C.Y., Zheng Q., Wu Z.L. (2022). Programmable Morphing Hydrogels for Soft Actuators and Robots: From Structure Designs to Active Functions. Accounts Chem. Res..

[3] Chen L.Z., Liu F.F., Abdiryim T., Liu X. (2024). Stimuli-responsive hydrogels as promising platforms for soft actuators. Mater. Today Phys..

[4] Ionov L. (2014). Hydrogel-based actuators: Possibilities and limitations. Mater. Today.

[5] Zhang X., Aziz S., Salahuddin B., Zhu Z. (2023). Thermoresponsive hydrogel artificial muscles. Matter.

[6] Ionov L. (2013). Biomimetic Hydrogel-Based Actuating Systems. Adv. Funct. Mater..

[7] Zhao X.H., Chen X.Y., Yuk H., Lin S.T., Liu X.Y., Parada G. (2021). Soft Materials by Design: Unconventional Polymer Networks Give Extreme Properties. Chem. Rev..

[8] Wang Y. (2018). Programmable hydrogels. Biomaterials.

[9] Wang H.H., Du J.L., Mao Y. (2025). Hydrogel-Based Continuum Soft Robots. Gels.

[10] Jayakumar A., Jose V.K., Lee J.M. (2020). Hydrogels for Medical and Environmental Applications. Small Methods.

[11] López-Díaz A., Vázquez A.S., Vázquez E. (2024). Hydrogels in Soft Robotics: Past, Present, and Future. ACS Nano.

[12] Caló E., Khutoryanskiy V.V. (2015). Biomedical applications of hydrogels: A review of patents and commercial products. Eur. Polym. J..

[13] Tan J.Y., Luo Y.N., Guo Y.Q., Zhou Y., Liao X.Y., Li D.X.L., Lai X.Y., Liu Y. (2023). Development of alginate-based hydrogels: Crosslinking strategies and biomedical applications. Int. J. Biol. Macromol..

[14] Yang J.S., Xie Y.J., He W. (2011). Research progress on chemical modification of alginate: A review. Carbohydr. Polym..

[15] Aksakal B., Kaplan Z., Turhan K. (2025). The influence of plasticizer on the mechanical, structural, thermal and strain recovery properties following stress-relaxation process of silk fibroin/sodium alginate biocomposites for biomedical applications. J. Mech. Behav. Biomed. Mater..

[16] Ren Y.Z., Wang Q., Xu W.L., Yang M.C., Guo W.H., He S.Q., Liu W.T. (2024). Alginate-based hydrogels mediated biomedical applications: A review. Int. J. Biol. Macromol..

[17] King A.H. (2019). Brown seaweed extracts (alginates). Food hydrocolloids.

[18] Leong J.Y., Lam W.H., Ho K.W., Voo W.P., Lee M.F.X., Lim H.P., Lim S.L., Tey B.T., Poncelet D., Chan E.S. (2016). Advances in fabricating spherical alginate hydrogels with controlled particle designs by ionotropic gelation as encapsulation systems. Particuology.

[19] Tan W.H., Takeuchi S. (2007). Monodisperse alginate hydrogel microbeads for cell encapsulation. Adv. Mater..

[20] Song W.L., Lima A.C., Mano J.F. (2010). Bioinspired methodology to fabricate hydrogel spheres for multi-applications using superhydrophobic substrates. Soft Matter.

[21] Sun R., Gao S., Zhang K., Cheng W.-T., Hu G. (2024). Recent advances in alginate-based composite gel spheres for removal of heavy metals. Int. J. Biol. Macromol..

[22] Dong Y.X., Ramey-Ward A.N., Salaita K. (2021). Programmable Mechanically Active Hydrogel-Based Materials. Adv. Mater..

[23] He Y., Tang J., Hu Y., Yang S., Xu F., Zrínyi M., Chen Y.M. (2023). Magnetic hydrogel-based flexible actuators: A comprehensive review on design, properties, and applications. Chem. Eng. J..

[24] Ouyang Y., Huang G.S., Cui J.Z., Zhu H., Yan G.H., Mei Y.F. (2022). Advances and Challenges of Hydrogel Materials for Robotic and Sensing Applications. Chem. Mater..

[25] Wang Z.H., Li W.J., Li C., Klingner A., Pei Y.T., Misra S., Khalil I.S.M. (2024). Magnetic alginate microrobots with dual-motion patterns through centrifugally driven flow control. Mater. Des..

[26] Chen X., Tian C.Y., Zhang H., Xie H. (2024). Magnetic-actuated hydrogel microrobots with multimodal motion and collective behavior. J. Mater. Chem. B.

[27] Li B.-Y., Lin T.-Y., Lai Y.-J., Chiu T.-H., Yeh Y.-C. (2025). Engineering Multiresponsive Alginate/PNIPAM/Carbon Nanotube Nanocomposite Hydrogels as On-Demand Drug Delivery Platforms. Small.

[28] Ching S.H., Bansal N., Bhandari B. (2017). Alginate gel particles-A review of production techniques and physical properties. Crit. Rev. Food Sci..

[29] Wang Y.T., Shen Z.P., Wang H.L., Song Z.P., Yu D.H., Li G.D., Liu X.N., Liu W.X. (2025). Progress in Research on Metal Ion Crosslinking Alginate-Based Gels. Gels.

[30] Cao L.Q., Lu W., Mata A., Nishinari K., Fang Y.P. (2020). Egg-box model-based gelation of alginate and pectin: A review. Carbohydr. Polym..

[31] Lee B.B., Ravindra P., Chan E.S. (2013). Size and Shape of Calcium Alginate Beads Produced by Extrusion Dripping. Chem. Eng. Technol..

[32] Mohamed M.G.A., Ambhorkar P., Samanipour R., Yang A., Ghafoor A., Kim K. (2020). Microfluidics-based fabrication of cell-laden microgels. Biomicrofluidics.

[33] Zhang C., Grossier R., Candoni N., Veesler S. (2021). Preparation of alginate hydrogel microparticles by gelation introducing cross-linkers using droplet-based microfluidics: A review of methods. Biomater. Res..

[34] Oveysi M., Zaker M.A., Peregrino G., Bazargan V., Marengo M. (2023). Droplet-based fabrication of alginate hydrogel microparticles in presence of surfactants. Microfluid. Nanofluid.

[35] Liu H.X., Wang C.Y., Gao Q.X., Liu X.X., Tong Z. (2008). Fabrication of novel core-shell hybrid alginate hydrogel beads. Int. J. Pharm..

[36] Yu L.F., Ni C., Grist S.M., Bayly C., Cheung K.C. (2015). Alginate core-shell beads for simplified three-dimensional tumor spheroid culture and drug screening. Biomed. Microdevices.

[37] Bennacef C., Desobry-Banon S., Probst L., Desobry S. (2023). Alginate Core-Shell Capsules Production through Coextrusion Methods: Principles and Technologies. Mar. Drugs.

[38] Zhu T.F., Wan L., Li R.Q., Zhang M., Li X.L., Liu Y.L., Cai D.J., Lu H.B. (2024). Janus structure hydrogels: Recent advances in synthetic strategies, biomedical microstructure and (bio)applications. Biomater. Sci..

[39] Zhao L.B., Pan L., Zhang K., Guo S.S., Liu W., Wang Y., Chen Y., Zhao X.Z., Chan H.L.W. (2009). Generation of Janus alginate hydrogel particles with magnetic anisotropy for cell encapsulation. Lab A Chip.

[40] Hwang S., Kwak B.K., Lee J., Kim D.S., Chang S.T., Park J., Lee J. (2012). Janus hydrogel particles and their aggregation behavior. Macromol. Res..

[41] Schmidt B.V.K.J. (2022). Multicompartment Hydrogels. Macromol. Rapid Commun..

[42] Håti A.G., Arnfinnsdottir N.B., Ostevold C., Sletmoen M., Etienne G., Amstad E., Stokke B.T. (2016). Microarrays for the study of compartmentalized microorganisms in alginate microbeads and (W/O/W) double emulsions. RSC Adv..

[43] Ciarleglio G., Placido M., Toto E., Santonicola M.G. (2024). Dual-Responsive Alginate/PNIPAM Microspheres Fabricated by Microemulsion-Based Electrospray. Polymers.

[44] Luo X., Su P., Zhang W., Raston C.L. (2019). Microfluidic Devices in Fabricating Nano or Micromaterials for Biomedical Applications. Adv. Mater. Technol..

[45] Mazzitelli S., Bottaro E., Nastruzzi C. (2017). Fabrication of Multifunctional Materials for Cell Transplantation by Microfluidics. RSC Smart Mater..

[46] Hinojosa-Ventura G., Acosta-Cuevas J.M., Velázquez-Carriles C.A., Navarro-López D.E., López-Alvarez M.Á., Ortega-de la Rosa N.D., Silva-Jara J.M. (2025). From Basic to Breakthroughs: The Journey of Microfluidic Devices in Hydrogel Droplet Generation. Gels.

[47] Zhu H.Y., Fu Y.Q., Jiang R., Yao J., Xiao L., Zeng G.M. (2014). Optimization of Copper(II) Adsorption onto Novel Magnetic Calcium Alginate/Maghemite Hydrogel Beads Using Response Surface Methodology. Ind. Eng. Chem. Res..

[48] Cholewinski A., Yang F.K., Zhao B.X. (2017). Underwater Contact Behavior of Alginate and Catechol-Conjugated Alginate Hydrogel Beads. Langmuir.

[49] Lekka M., Sainz-Serp D., Kulik A.J., Wandrey C. (2004). Hydrogel microspheres: Influence of chemical composition on surface morphology, local elastic properties, and bulk mechanical characteristics. Langmuir.

[50] Salahuddin B., Warren H., Spinks G.M. (2020). Thermally actuated hydrogel bead based braided artificial muscle. Smart Mater. Struct..

[51] Zhang H.J., Zhang Y.A., He L.F., Yang B.A., Zhu S.J., Yao M.H. (2016). Thermal-responsive poly(N-isopropyl acrylamide)/sodium alginate hydrogels: Preparation, swelling behaviors, and mechanical properties. Colloid Polym. Sci..

[52] Zhang Q., Li J.W., Qu Q.D., Pan S., Yu K.Y., Liu Y.S. (2024). Graphene oxide modified sodium alginate/polyethylene glycol phase change material hydrogel scaffold composite with photothermal temperature control for potential bone tissue regeneration. J. Mater. Res. Technol..

[53] Gan L., Li H., Chen L.W., Xu L.J., Liu J., Geng A.B., Mei C.T., Shang S.M. (2018). Graphene oxide incorporated alginate hydrogel beads for the removal of various organic dyes and bisphenol A in water. Colloid Polym. Sci..

[54] Jiang Y.H., Wang Y., Li Q., Yu C., Chu W.L. (2020). Natural Polymer-based Stimuli-responsive Hydrogels. Curr. Med. Chem..

[55] Sangwan W., Petcharoen K., Paradee N., Lerdwijitjarud W., Sirivat A. (2016). Electrically responsive materials based on polycarbazole/sodium alginate hydrogel blend for soft and flexible actuator application. Carbohydr. Polym..

[56] Zheng D., Wang K., Bai B., Hu N., Wang H. (2022). Swelling and glyphosate-controlled release behavior of multi-responsive alginate-gP (NIPAm-co-NDEAm)-based hydrogel. Carbohydr. Polym..

[57] Mair L.O., Chowdhury S., Paredes-Juarez G.A., Guix M., Bi C., Johnson B., English B.W., Jafari S., Baker-McKee J., Watson-Daniels J. (2019). Magnetically aligned nanorods in alginate capsules (MANiACs): Soft matter tumbling robots for manipulation and drug delivery. Micromachines.

[58] Tirella A., Magliaro C., Penta M., Troncone M., Pimentel R., Ahluwalia A. (2014). Sphyga: A multiparameter open source tool for fabricating smart and tunable hydrogel microbeads. Biofabrication.

[59] Alshehri A.M., Wilson O.C., Dahal B., Philip J., Luo X.L., Raub C.B. (2017). Magnetic nanoparticle-loaded alginate beads for local micro-actuation of tissue constructs. Colloid Surface B.

[60] Salahuddin B., Aziz S., Gao S., Hossain M.S.A., Billah M., Zhu Z.H., Amiralian N. (2022). Magnetic Hydrogel Composite for Wastewater Treatment. Polymers.

[61] Majumdar S., Krishnatreya G., Gogoi N., Thakur D., Chowdhury D. (2016). Carbon-Dot-Coated Alginate Beads as a Smart Stimuli-Responsive Drug Delivery System. ACS Appl. Mater. Interfaces.

[62] Tiwari A., Gajbhiye V., Jain A., Verma A., Shaikh A., Salve R., Jain S.K. (2022). Hyaluronic acid functionalized liposomes embedded in biodegradable beads for duo drugs delivery to oxaliplatin-resistant colon cancer. J. Drug Deliv. Sci. Technol..

[63] Roquero D.M., Katz E. (2022). “Smart” alginate hydrogels in biosensing, bioactuation and biocomputing: State-of-the-art and perspectives. Sens. Actuators Rep..

[64] Lu L., Zhao H., Lu Y., Zhang Y., Wang X., Fan C., Li Z., Wu Z. (2024). Design and Control of the Magnetically Actuated Micro/Nanorobot Swarm toward Biomedical Applications. Adv. Healthc. Mater..

[65] Maity C., Das N. (2022). Alginate-Based Smart Materials and Their Application: Recent Advances and Perspectives. Top. Curr. Chem..

[66] Swamy B.Y., Chang J.H., Ahn H., Lee W.K., Chung I. (2013). Thermoresponsive N-vinyl caprolactam grafted sodium alginate hydrogel beads for the controlled release of an anticancer drug. Cellulose.

[67] Lencina M.M.S., Iatridi Z., Villar M.A., Tsitsilianis C. (2014). Thermoresponsive hydrogels from alginate-based graft copolymers. Eur. Polym. J..

[68] Liu M., Wen Y.T., Song X., Zhu J.L., Li J. (2019). A smart thermoresponsive adsorption system for efficient copper ion removal based on alginate-poly(-isopropylacrylamide) graft copolymer. Carbohydr. Polym..

[69] Zhang J.N., Cui Z.F., Field R., Moloney M.G., Rimmer S., Ye H. (2015). Thermo-responsive microcarriers based on poly(-isopropylacrylamide). Eur. Polym. J..

[70] Degen P., Leick S., Siedenbiedel F., Rehage H. (2012). Magnetic switchable alginate beads. Colloid Polym. Sci..

[71] Fu Y., Yao J.J., Zhao H.H., Zhao G., Wan Z.S., Guo R.Z. (2020). A muscle-like magnetorheological actuator based on bidisperse magnetic particles enhanced flexible alginate-gelatin sponges. Smart Mater. Struct..

[72] Gunatilake U.B., Venkatesan M., Basabe-Desmonts L., Benito-Lopez F. (2022). and u Magnetic Phase Synthesised Magneto-Driven Alginate Beads. J. Colloid Interface Sci..

[73] Yang J., Wang S., Yao J., Wei K., Yu T., Fang M., Jiang Z. (2023). Electrically actuated characteristics and electrochemical mechanism of a flexible biomimetic artificial muscle regulated by the responsive polymer of the calcium alginate gelation. Polym. Test..

[74] Palza H., Zapata P.A., Angulo-Pineda C. (2019). Electroactive Smart Polymers for Biomedical Applications. Materials.

[75] Gehlen D.B. (2021). Microgel-based Regenerative Materials and Biofunctionalization. Doctoral Dissertation.

[76] Appiah C., Arndt C., Siemsen K., Heitmann A., Staubitz A., Selhuber-Unkel C. (2019). Living Materials Herald a New Era in Soft Robotics. Adv. Mater..

[77] Tordi P., Tamayo A., Jeong Y., Bonini M., Samorì P. (2024). Multiresponsive Ionic Conductive Alginate/Gelatin Organohydrogels with Tunable Functions. Adv. Funct. Mater..

[78] Das A., Babu A., Chakraborty S., Van Guyse J.F.R., Hoogenboom R., Maji S. (2024). Poly(-isopropylacrylamide) and Its Copolymers: A Review on Recent Advances in the Areas of Sensing and Biosensing. Adv. Funct. Mater..

[79] Hanyková L., Stastná J., Krakovsky I. (2024). Responsive Acrylamide-Based Hydrogels: Advances in Interpenetrating Polymer Structures. Gels.

[80] Deng Z.X., Yu R., Guo B.L. (2021). Stimuli-responsive conductive hydrogels: Design, properties, and applications. Mater. Chem. Front..

[81] Liu Y.Z., Chen Z., Xu J.H. (2024). Recent advances in the microfluidic generation of shape-controllable hydrogel microparticles and their applications. Green Chem. Eng..

[82] Shang J.J., Le X.X., Zhang J.W., Chen T., Theato P. (2019). Trends in polymeric shape memory hydrogels and hydrogel actuators (vol 10, pg 1036, 2019). Polym. Chem..

[83] Hu L., Wan Y., Zhang Q., Serpe M.J. (2020). Harnessing the Power of Stimuli-Responsive Polymers for Actuation. Adv. Funct. Mater..

[84] Zhang X., Aziz S., Zhu Z.H. (2025). Tough and Fast Thermoresponsive Hydrogel Soft Actuators. Adv. Mater. Technol..

[85] Zhang X., Aziz S., Salahuddin B., Zhu Z.H. (2024). Bioinspired Hydro- and Hydrothermally Responsive Tubular Soft Actuators. ACS Appl. Mater. Interfaces.

[86] Aziz S., Zhang X., Naficy S., Salahuddin B., Jager E.W.H., Zhu Z.H. (2023). Plant-Like Tropisms in Artificial Muscles. Adv. Mater..

[87] Park J., Guan W.X., Yu G.H. (2025). Smart Hydrogels for Sustainable Agriculture. EcoMat.

[88] Mao S.D., Johir M.A., Onggowarsito C., Feng A., Nghiem L.D., Fu Q. (2022). Recent developments of hydrogel based solar water purification technology. Mater. Adv..

[89] Sroka K., Sroka P. (2024). Superabsorbent Hydrogels in the Agriculture and Reclamation of Degraded Areas. Sustainability.

[90] Chung H.J., Parsons A.M., Zheng L.L. (2021). Magnetically Controlled Soft Robotics Utilizing Elastomers and Gels in Actuation: A Review. Adv. Intell. Syst..

[91] Ebrahimi N., Bi C.H., Cappelleri D.J., Ciuti G., Conn A.T., Faivre D., Habibi N., Hosovsky A., Iacovacci V., Khalil I.S.M. (2021). Magnetic Actuation Methods in Bio/Soft Robotics. Adv. Funct. Mater..

[92] Liu Y., Lin G.G., Medina-Sánchez M., Guix M., Makarov D., Jin D.Y. (2023). Responsive Magnetic Nanocomposites for Intelligent Shape-Morphing Microrobots. ACS Nano.

[93] Philippova O., Barabanova A., Molchanov V., Khokhlov A. (2011). Magnetic polymer beads: Recent trends and developments in synthetic design and applications. Eur. Polym. J..

[94] Fusco S., Sakar M.S., Kennedy S., Peters C., Pane S., Mooney D., Nelson B.J. Self-folding mobile microrobots for biomedical applications. Proceedings of the 2014 IEEE International Conference on Robotics and Automation (ICRA).

[95] Chen Q., Wu S. (2025). Stimuli-Responsive Polymers for Tubal Actuators. Chem. Eur. J..

[96] Incel A., Demir M.M. (2018). Triboluminescent composite microspheres consisting of alginate and EuD TEA crystals. Sens. Actuators A Phys..

[97] Habib M., Berthalon S., Leclercq L., Tourrette A., Sharkawi T., Blanquer S. (2024). Dual Cross-Linked Stimuli-Responsive Alginate-Based Hydrogels. Biomacromolecules.

[98] Tordi P., Ridi F., Samori P., Bonini M. (2025). Cation-Alginate Complexes and Their Hydrogels: A Powerful Toolkit for the Development of Next-Generation Sustainable Functional Materials. Adv. Funct. Mater..

[99] Banerjee H., Suhail M., Ren H.L. (2018). Hydrogel Actuators and Sensors for Biomedical Soft Robots: Brief Overview with Impending Challenges. Biomimetics.

[100] Bercea M. (2022). Bioinspired Hydrogels as Platforms for Life-Science Applications: Challenges and Opportunities. Polymers.

[101] Buenger D., Topuz F., Groll J. (2012). Hydrogels in sensing applications. Prog. Polym. Sci..

[102] Liu Y.Z., Tottori N., Nisisako T. (2019). Microfluidic synthesis of highly spherical calcium alginate hydrogels based on external gelation using an emulsion reactant. Sens. Actuators B Chem..

[103] Li T., Yu S., Sun B., Li Y., Wang X., Pan Y., Song C., Ren Y., Zhang Z., Grattan K.T.V. (2023). Bioinspired claw-engaged and biolubricated swimming microrobots creating active retention in blood vessels. Sci. Adv..

[104] Moe S.T., Skjaak-Braek G., Elgsaeter A., Smidsroed O. (1993). Swelling of covalently crosslinked alginate gels: Influence of ionic solutes and nonpolar solvents. Macromolecules.

[105] Lee K.Y., Rowley J.A., Eiselt P., Moy E.M., Bouhadir K.H., Mooney D.J. (2000). Controlling mechanical and swelling properties of alginate hydrogels independently by cross-linker type and cross-linking density. Macromolecules.

[106] Salahuddin B.B. (2020). Hydrogel Based Braided Artificial Muscles.

[107] Supramaniam J., Adnan R., Kaus N.H.M., Bushra R. (2018). Magnetic nanocellulose alginate hydrogel beads as potential drug delivery system. Int. J. Biol. Macromol..

[108] Joshi A., Solanki S., Chaudhari R., Bahadur D., Aslam M., Srivastava R. (2011). Multifunctional alginate microspheres for biosensing, drug delivery and magnetic resonance imaging. Acta Biomater..

[109] Li J.Y., Li X.J., Luo T., Wang R., Liu C.C., Chen S.X., Li D.F., Yue J.B., Cheng S.H., Sun D. (2018). Development of a magnetic microrobot for carrying and delivering targeted cells. Sci. Robot..

[110] Singh A., Kar A.K., Singh D., Verma R., Shraogi N., Zehra A., Gautam K., Anbumani S., Ghosh D., Patnaik S. (2022). pH-responsive eco-friendly chitosan modified cenosphere/alginate composite hydrogel beads as carrier for controlled release of Imidacloprid towards sustainable pest control. Chem. Eng. J..

[111] Ahmad A., Ahmad I., Kamal T., Asiri A.M., Tabassum S. (2022). Sodium Alginate-Based Nanomaterials for Wastewater Treatment.

[112] Li K., Sun J.H., He S.P., Zhou X.X., Li H.Y., Liu Y.X. (2022). On-demand preparation of calcium alginate microspheres via piezoelectric microfluidics. Sens. Actuators A Phys..

[113] Ding F., Zhang L., Chen X., Yin W., Ni L., Wang M. (2022). Photothermal nanohybrid hydrogels for biomedical applications. Front. Bioeng. Biotechnol..

[114] Cui X., Ruan Q., Zhuo X., Xia X., Hu J., Fu R., Li Y., Wang J., Xu H. (2023). Photothermal Nanomaterials: A Powerful Light-to-Heat Converter. Chem. Rev..

[115] van der Sman R.G.M. (2015). Biopolymer gel swelling analysed with scaling laws and Flory–Rehner theory. Food Hydrocoll..

[116] Malektaj H., Drozdov A.D., deClaville Christiansen J. (2023). Mechanical Properties of Alginate Hydrogels Cross-Linked with Multivalent Cations. Polymers.

[117] Abroug N., Schöbel L., Boccaccini A.R., Seitz H. (2024). Quantitative Macromolecular Modeling Assay of Biopolymer-Based Hydrogels. Gels.

[118] Lee P.I. (1985). Kinetics of drug release from hydrogel matrices. J. Control. Release.

[119] Jastram A., Lindner T., Luebbert C., Sadowski G., Kragl U. (2021). Swelling and Diffusion in Polymerized Ionic Liquids-Based Hydrogels. Polymers.

[120] Bertos G.A., Papadopoulos E.G., Segil J. (2019). Chapter Six—Upper-Limb Prosthetic Devices. Handbook of Biomechatronics.

[121] Yang C., Wang W., Yao C., Xie R., Ju X.-J., Liu Z., Chu L.-Y. (2015). Hydrogel Walkers with Electro-Driven Motility for Cargo Transport. Sci. Rep..

[122] Shen S., Qiu J., Huo D., Xia Y. (2024). Nanomaterial-Enabled Photothermal Heating and Its Use for Cancer Therapy via Localized Hyperthermia. Small.

[123] Zhuang J., Jia L., Li C., Yang R., Wang J., Wang W.-a., Zhou H., Luo X. (2025). Recent advances in photothermal nanomaterials for ophthalmic applications. Beilstein J. Nanotechnol..

[124] Yang Y., Kirmizitas F.C., Sokolich M., Valencia A., Rivas D., Karakan M.Ç., White A.E., Malikopoulos A.A., Das S. Rolling Helical Microrobots for Cell Patterning. Proceedings of the 2023 International Conference on Manipulation, Automation and Robotics at Small Scales (MARSS).

[125] Zhou H., Mayorga-Martinez C.C., Pané S., Zhang L., Pumera M. (2021). Magnetically Driven Micro and Nanorobots. Chem. Rev..

[126] Xu T., Yu J., Yan X., Choi H., Zhang L. (2015). Magnetic Actuation Based Motion Control for Microrobots: An Overview. Micromachines.

[127] Ahmadinejad M., Marshall J.S. (2024). Magnetic nanoparticle interaction with a hydrogel in an oscillating magnetic field. Phys. Fluids.

[128] Jiang L., Yang W., Xie L., Liu Y., Wang X., Wu X., Zhou F., Hu H. (2023). Experimental study on mechanism of stable drag reduction with hydrogel interface. Tribol. Int..

[129] Zang J., Liu F. (2008). Modified Timoshenko formula for bending of ultrathin strained bilayer films. Appl. Phys. Lett..

[130] Kim J., Kim C., Song Y., Jeong S.-G., Kim T.-S., Lee C.-S. (2017). Reversible self-bending soft hydrogel microstructures with mechanically optimized designs. Chem. Eng. J..

[131] Scalet G. (2024). Programmable materials: Current trends, challenges, and perspectives. Appl. Mater. Today.

[132] Xu K., Yuan G., Zheng J., Zhang Y., Wang J., Guo H. (2024). Bioinspired microrobots and their biomedical applications. Nanoscale.

[133] Li J., Esteban-Fernández de Ávila B., Gao W., Zhang L., Wang J. (2017). Micro/nanorobots for biomedicine: Delivery, surgery, sensing, and detoxification. Sci. Robot..

[134] Salahuddin B., Aziz S., Zhang X., Feng D., Zhu Z. (2024). Biomorph Soft Actuators with Tardigrade-Like Resilience. Adv. Funct. Mater..

[135] Tian Y., Han W., Yeung K.L. (2023). Magnetic Microsphere Scaffold-Based Soft Microbots for Targeted Mesenchymal Stem Cell Delivery. Small.

[136] Jeong C., Kim S., Lee C., Cho S., Kim S.-B. (2020). Changes in the Physical Properties of Calcium Alginate Gel Beads under a Wide Range of Gelation Temperature Conditions. Foods.

[137] Schneider F., Draheim J., Kamberger R., Wallrabe U. (2009). Process and material properties of polydimethylsiloxane (PDMS) for Optical MEMS. Sens. Actuators A Phys..

[138] Zhou B., Aouraghe M.A., Chen W., Jiang Q., Xu F. (2023). Highly Responsive Soft Electrothermal Actuator with High-Output Force Based on Polydimethylsiloxane (PDMS)-Coated Carbon Nanotube (CNT) Sponge. Nano Lett..

[139] Aziz S., Spinks G.M. (2020). Torsional artificial muscles. Mater. Horiz..

[140] Aziz S., Villacorta B., Naficy S., Salahuddin B., Gao S., Baigh T.A., Sangian D., Zhu Z. (2021). A microwave powered polymeric artificial muscle. Appl. Mater. Today.

[141] Peng S., Cao X., Sun Y., Chen L., Ma C., Yang L., Zhao H., Liu Q., Liu Z., Ma C. (2023). Polyurethane Shape Memory Polymer/pH-Responsive Hydrogel Hybrid for Bi-Function Synergistic Actuations. Gels.

[142] Pringpromsuk S., Xia H., Ni Q.-Q. (2020). Multifunctional stimuli-responsive shape memory polyurethane gels for soft actuators. Sens. Actuators A Phys..

